# Somatosensory Loss Influences the Adoption of Self-Centered Versus Decentered Perspectives

**DOI:** 10.3389/fpsyg.2019.00419

**Published:** 2019-03-11

**Authors:** Gabriel Arnold, Fabrice R. Sarlegna, Laura G. Fernandez, Malika Auvray

**Affiliations:** ^1^ Caylar, Villebon-sur-Yvette, France; ^2^ Institut des Systèmes Intelligents et de Robotique (ISIR), CNRS UMR 7222, Sorbonne Université, Paris, France; ^3^ Aix Marseille Univ, CNRS, ISM, Marseille, France

**Keywords:** body and self, proprioception, spatial perspectives, somatosensory loss, individual differences

## Abstract

The body and the self are commonly experienced as forming a unity. Experiencing the external world as distinct from the self and the body strongly relies on adopting a single self-centered perspective which results in integrating multisensory sensations into one egocentric body-centered reference frame. Body posture and somatosensory representations have been reported to influence perception and specifically the reference frame relative to which multisensory sensations are coded. In the study reported here, we investigated the role of somatosensory and visual information in adopting self-centered and decentered spatial perspectives. Two deafferented patients who have neither tactile nor proprioceptive perception below the head and a group of age-matched control participants performed a graphesthesia task, consisting of the recognition of ambiguous letters (b, d, p, and q) drawn tactilely on head surfaces. To answer which letter was drawn, the participants can adopt either a self-centered perspective or a decentered one (i.e., centered on a body part or on an external location). The participants’ responses can be used, in turn, to infer the way the left-right and top-bottom letters’ axes are assigned with respect to the left-right and top-bottom axes of their body. In order to evaluate the influence of body posture, the ambiguous letters were drawn on the participants’ forehead, left, and right surfaces of the head, with the head aligned or rotated in yaw relative to the trunk. In order to evaluate the role of external information, the participants completed the task with their eyes open in one session and closed in another one. The results obtained in control participants revealed that their preferred perspective varied with body posture but not with vision. Different results were obtained with the deafferented patients who overall do not show any significant effect of their body posture on their preferred perspective. This result suggests that the orientation of their self is not influenced by their physical body. There was an effect of vision for only one of the two patients. The deafferented patients rely on strategies that are more prone to interindividual differences, which highlights the crucial role of somatosensory information in adopting self-centered spatial perspectives.

## Introduction

One of the key roles of bodily self-consciousness consists in experiencing the body and the self as forming a unity. The experiential self, the locus of our sensations and commands of action, is typically felt as being located within the body and as being delimited by the boundaries of the body (see De Vignemont, 2018, for analyses of the concepts of bodily self-awareness and bodily ownership). The self can also be subjectively located within one specific body part, predominantly the head or the trunk (Bertossa et al., 2008; Limanowski and Hecht, 2011; Alsmith and Longo, 2014). In addition, experiencing the external world as distinct from the self strongly relies on integrating external multisensory sensations (e.g., visual, auditory, tactile) and internal somatosensory sensations into one egocentric body-centered reference frame, which results in perceiving single objects as being located at specific locations relative to the body. For instance, the simultaneous appearance of a car in the left visual field and the hearing of an engine noise in the left auditory field commonly result in a unique percept of one single moving car located leftward to the body.

**Figure 1:**
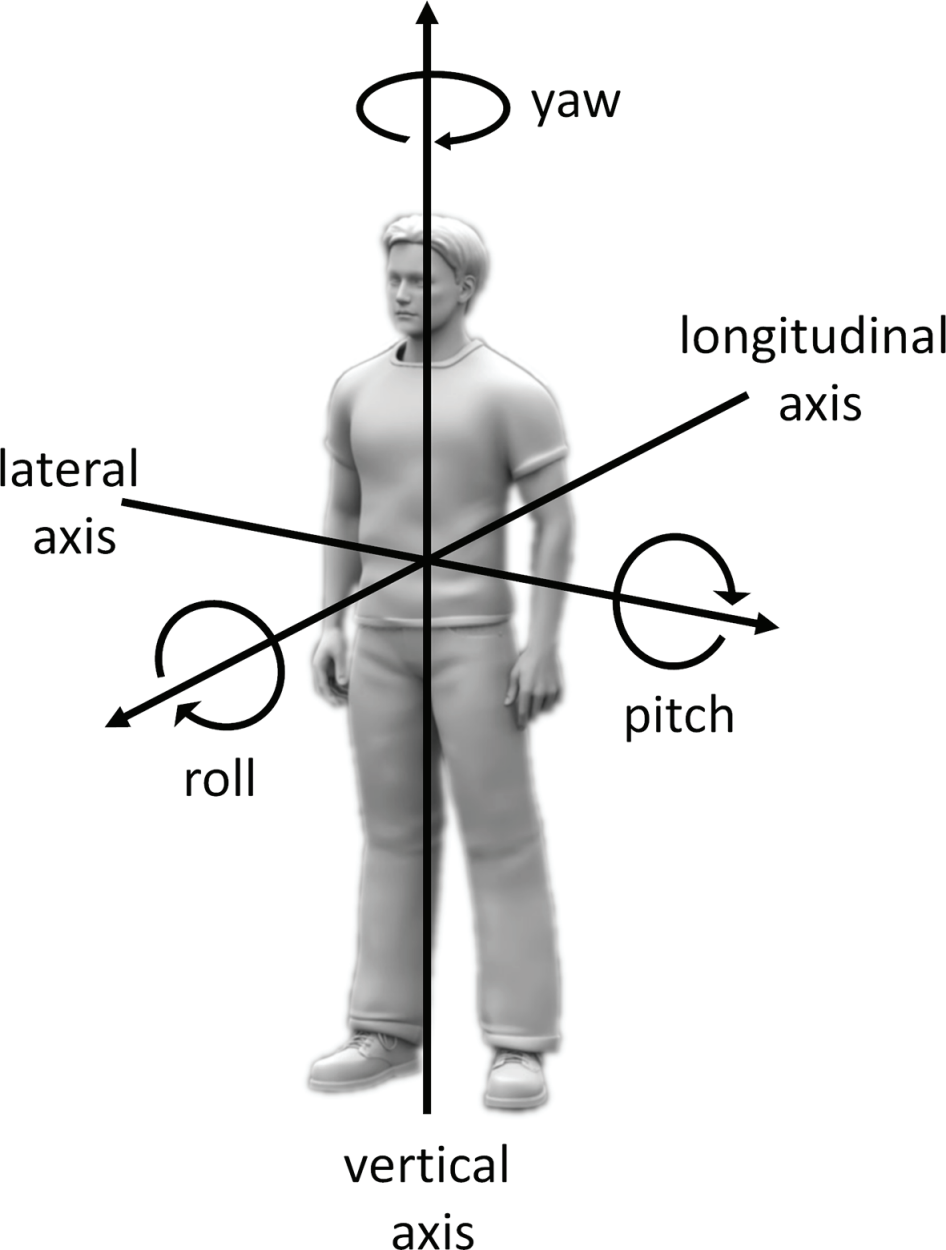
Definitions of the axes of body rotations. For full-body rotations, axes of rotation can be defined relative to a reference frame considering an upright body relative to gravity with the head straight ahead. According to this reference frame, rotations in roll, pitch, and yaw correspond to rotations around the longitudinal (back to front), lateral (left to right), and vertical (foot to head) body axes, respectively. For rotations of specific body parts (e.g., the head), axes of rotation can be defined relative to the same reference frame but with an additional reference to the body part relative to which the moving body part rotates. For instance, turning the head toward the left shoulder when keeping an upright posture relative to gravity can be defined as rotating the head in yaw relative to the trunk.

The multisensory integration of external and internal information into a common body-centered reference frame is thought to rely on the adoption of a single self-centered spatial perspective (Blanke, 2012; Arnold et al., 2017). However, in the same way, as the body posture influences the perception of visual, auditory, and tactile sensations, body posture can also influence the definition of the egocentric reference frame into which these perceptions are integrated (Harris et al., 2015). For instance, deciding whether an object is oriented with its top up and its bottom down, which can be called perceptual upright, requires integrating visual and somatosensory cues. This process has been reported to be influenced by full-body rotations in roll or in pitch relative to gravity (Dyde et al., 2006; see Figure 1, for definitions of body rotation axes). In addition, egocentric and allocentric judgments of verticality have been reported to rely both on visual and somatosensory cues, with however a greater weight given to body reference for egocentric judgments such as indicating the vertical axis of our own head (Barnett-Cowan and Harris, 2008).

When standing upright with the head straight ahead as in Figure 1, there is little ambiguity when deciding where are the left, right, top, bottom, front, and back of the body. However, ambiguities appear when the different body parts are not aligned. Misalignments of the head and trunk, such as when the head is rotated in yaw relative to the trunk or bended forward, create left-right or top-bottom ambiguities, which have been reported to bias perception. For instance, when the head is rotated in yaw relative to the trunk, localization judgments of tactile stimulation on the trunk are biased toward the direction of the head (Ho and Spence, 2007; Pritchett et al., 2012). Gaze orientation, which consists of the combination of head and eye orientation, has also been reported to bias touch localization (Harrar and Haris, 2010). This influence of body posture and specifically the influence of head or gaze orientation on touch can reflect the existence of a reference frame transformation, from a body to a visual reference frame. A gaze-based visual reference frame would be particularly adapted for multisensory integration during perception and action (Cohen and Andersen, 2002; Harris et al., 2015). The importance of reference frame transformation into a unified head-centered or gaze-centered reference frame also reflects the important role of the head in defining the self. The use of a unified head-centered perspective allows the observer to perceive a unified external world, distinct from the self (Arnold et al., 2017).

Regarding spatial perspectives, when judging whether an object is located to the left or to the right of another person who has an ambiguous body posture (i.e., head rotated in yaw relative to the trunk), the reference frame used to make left-right judgments has been reported to result on a weighted combination of the person’s head and trunk reference frames (Alsmith et al., 2017). The influence of body posture on the location and orientation of the self (Alsmith and Longo, 2014; Alsmith et al., 2017) and on other spatial processes, such as mental rotation (Amorim et al., 2006) or perspective-taking (Kessler and Thomson, 2010; Arnold and Auvray, 2017), has indeed been described to reflect the involvement of embodied processes. According to this view, spatial cognition involves not only spatial representations but also motor and somatosensory representations of the body (Renault et al., 2018). More specifically, mentally displacing the self to adopt a decentered perspective would involve both a mental change in body posture and an emulation of the movements that would be necessary to physically place the body in a novel position and orientation.

Given the important role of somatosensory information and somatosensory representations in spatial cognition, what happens when bodily sensations are deficient? Previous studies have reported that somatosensory loss has profound consequences on spatial cognition in two rare cases of massive yet selective deafferentation. These two patients have lost proprioceptive and tactile afferents from below the neck (IW) and from the nose down (GL) due to a sensory neuropathy (for a more elaborate description of the patients, see Cole and Paillard, 1995; Miall et al., 2018). First, somatosensory loss has been reported to affect judgments of self-orientation as well as object orientation (Bringoux et al., 2016). For instance, to judge the orientation of external objects relative to gravity, GL is more influenced by visual surrounding than controls in a classic rod-and-frame test (Oltman, 1968) in which participants have to align a rod with the gravitational vertical. In addition, contrary to controls, GL is insensitive to self-rotation in pitch relative to gravity up to 18°. Second, somatosensory loss has been reported to impact imagery processes (Ter Horst et al., 2012). Compared to controls, IW has impaired motor imagery but enhanced visual imagery performance in mental rotation tasks. For instance, when judging the orientation of seen corporeal objects (e.g., hands rotated in roll relative to gravity), contrary to controls, IW’s mental rotation processes are not influenced by the orientation of his own hands, suggesting the use of a visual strategy, rather than a motor one. Taken together, these results show that deafferented patients differ from controls in spatial cognition, both with respect to the used perceptual cues (which is obvious considering the patients’ somatosensory loss) and to the individual strategies that are involved (see also Renault et al., 2018).

**Figure 2:**
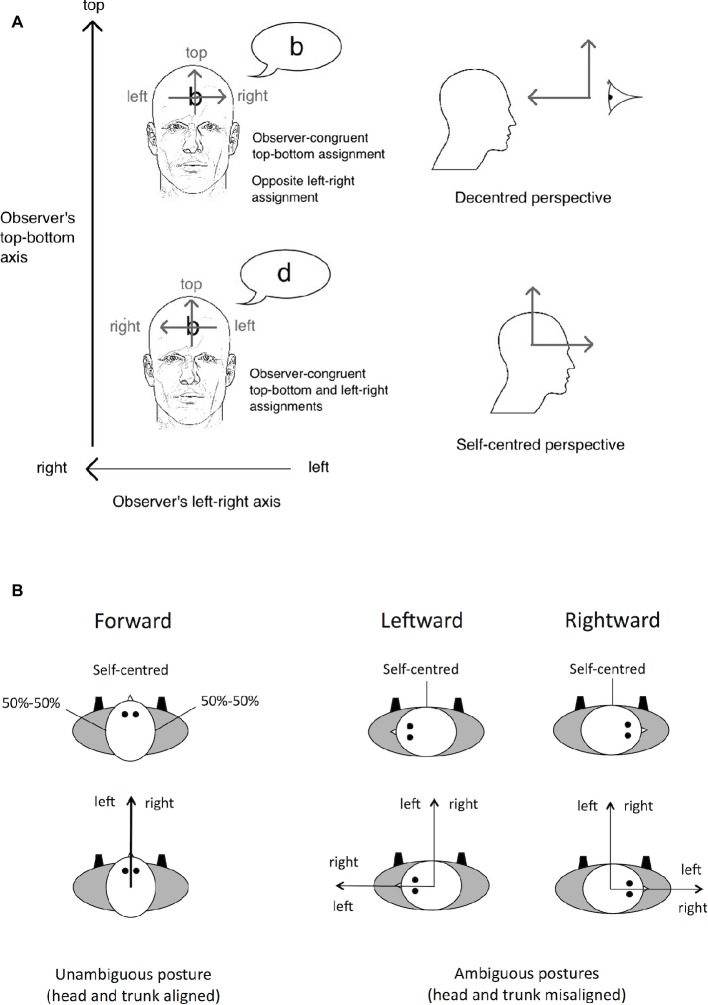
Illustration of the spatial perspectives – self-centered versus decentered – that can be adopted in the graphesthesia task. **(A)** When the letter is drawn on the participants’ forehead, some participants perceive the letter “b,” assigning the left-right axis of the letter in the direction opposite to their own head’s left-right axis. This assignment may result from a decentered perspective whose origin is in front of the participant’s head. Other participants will perceive the mirror-reversed letter “d” instead, assigning the left-right axis of the letter in the same direction as their own head’s left-right axis. This assignment may result from a self-centered perspective whose origin is located inside the head. *Source:* Arnold et al., 2017. **(B)** Illustration of the results reported by Natsoulas and Dubanovski (1964) showing that the adoption of self-centered versus decentered perspectives on the sides of the head depends on the orientation of the head in yaw relative to the trunk. With an ambiguous posture (head and trunk misaligned), the left-right axis of the observer’s egocentric reference frame may be assigned with respect to the head or the trunk. When a tactile letter is drawn on the side of the head, with such an ambiguous posture, the left-right axis of the letter is assigned with respect to the trunk. Figure 2A is reprinted from Consciousness and Cognition 56. Arnold, G., Spence, C., and Auvray, M.

In the present study, we investigated the role of somatosensory information and the impact of somatosensory loss when making spatial judgments directly relative to oneself. The graphesthesia task, which consists of recognizing tactile ambiguous letters (e.g., b, d, p, and q) drawn on the body surface, is an optimal tool to evaluate the spatial perspectives that are adopted to interpret tactile stimulation (Natsoulas and Dubanovski, 1964; Parsons and Shimojo, 1987; Sekiyama, 1991; Ferrè et al., 2014; for a review, see Arnold et al., 2017). When drawing ambiguous letters on the body surface, different spatial perspectives can be adopted, either self-centered (i.e., centered on one body part) or decentered (i.e., centered on a location external to the body). The participants’ responses can be used to infer the spatial perspective they have adopted. For instance, when the letter “b” is drawn on a participant’s forehead (from the experimenter’s viewpoint), the recognition of the letter “b” requires the participant to adopt the experimenter’s perspective, hence a decentered perspective. However, if the participant adopts a self-centered perspective, centered on the forehead, the letter may be recognized as the mirror-reversed letter “d,” as if the letter was mentally projected forward the participant (see Figure 2A).

Individual differences in the adoption of spatial perspectives have been reported. Most people spontaneously adopt a self-centered perspective, whereas some people adopt a decentered one (approximately 20% for the latter, Arnold et al., 2016). The adoption of spatial perspectives is also influenced by the physical body posture (Natsoulas and Dubanovski, 1964). For instance, when ambiguous letters are drawn on the left and right sides of the head, self-centered and decentered perspectives are adopted equally often when the head is oriented looking forward in the same direction as the trunk. However, the adoption of self-centered versus decentered perspectives varies with the orientation of the head in yaw relative to the trunk. Indeed, when the head is rotated leftward or rightward (i.e., toward the left or right shoulder), the sides of the head are aligned with the front of the trunk and people mostly adopt a self-centered perspective (see Figure 2B). Taken together, these results can be interpreted as reflecting the role of both trunk and head orientations in spatially defining the self relative to the body (see also O’Brien and Auvray, 2016, for the role of hand orientation on the adopted perspective).

The first aim of the present study was to evaluate the impact of somatosensory loss in adopting self-centered versus decentered perspectives. To do so, the performance of two well-characterized deafferented patients and 20 age-matched controls in the graphesthesia task was compared. Ambiguous letters (b, d, p, and q) were manually drawn on people’s forehead, left side, and right side of the head, with the head aligned or rotated in yaw relative to the trunk. For control participants, previous work made us expect that adopting one or the other perspective should be influenced by the orientation of the head in yaw relative to the trunk, specifically when the ambiguous letters are drawn on the sides of the head (Natsoulas and Dubanovski, 1964). More specifically, the self-centered perspective should be adopted more often on the left side when the head is oriented rightward rather than forward and on the right side when the head is oriented leftward rather than forward.

Following previous works on the impact of sensory loss on spatial cognition (Ter Horst et al., 2012; Bringoux et al., 2016), we hypothesized that, due to their massive sensory loss, the two deafferented patients’ responses should be less influenced by their body posture than controls. However, as their locus of somatosensory loss differ (from neck and from nose down), the two patients should differ in the influence of body posture on the adopted perspective. As the crucial manipulation in our experiment is the orientation of the head in yaw relative to the trunk, proprioception of the neck should play a specific role. For instance, neck proprioception has been reported to play a role in posture stability, allowing the central nervous system to consider misalignment between the head and trunk (Blouin et al., 2007). Consequently, as proprioception of the neck is preserved for IW but not for GL, IW should be more influenced by body posture than GL. Finally, considering that somatosensory sensation is crucial to perform egocentric judgments (Lackner, 1988; Bringoux et al., 2016), we also hypothesized that deafferented patients may preferentially rely on a decentered perspective. However, any preference in perspective is likely mediated by strategies developed as a function of individual characteristics (Arnold et al., 2017).

The second aim of the present study was to investigate the influence of visual information on the adoption of self-centered versus decentered perspectives to interpret tactile stimulation. Judgments of self-orientation rely both on visual and somatosensory cues (Dyde et al., 2006; Barnett-Cowan and Harris, 2008; Barnett-Cowan et al., 2010). In the graphesthesia task, adopting a decentered perspective can be considered as adopting the perspective of the experimenter who is drawing the tactile letter or more generally the perspective of another person who is facing the participant. The adoption of decentered perspectives has been reported to be influenced by the presence (Arnold et al., 2017) and position (Cohen and Lewin, 1986) of the experimenter. More generally, the presence of another person has been reported to influence to a large extent the tendency to adopt a decentered perspective (Tversky and Hard, 2009), even when the person is not relevant for the task (Quesque et al., 2018). In the present study, the participants completed the graphesthesia task both with their eyes open and their eyes closed, that is, seeing or not the experimenter. We expected the decentered perspective to be adopted more often when the eyes are open than when they are closed, in particular for the deafferented patients who are reported to rely more on visual information than control participants (Blouin et al., 1993; Bringoux et al., 2016).

## Materials and Methods

### Participants

Two deafferented participants with severe somatosensory loss (GL, a 70-year-old woman; IW, a 65-year-old man) and 20 age-matched control participants (mean age = 68.2 years, range = 60–78; 10 men and 10 women) completed the experiment. To summarize their impairment, GL and IW suffered from an acute sensory neuronopathy when they were 31 and 19 years old, respectively. This resulted in the specific loss of large-diameter myelinated afferents. Since then, they have lost all somatosensory modalities (kinesthesia, tendon reflexes, touch, vibration, and pressure) in their body from nose down for GL (trigeminal division 3) and from neck down for IW (C3 root level). Small sensory fiber functions, such as pain and temperature perception, were not affected and neither were the motor nerves. The somatosensory loss is massive in these two patients, and it results in severe motor deficit, as for instance, they both use a wheelchair and they are severely impaired in the absence of vision (Blouin et al., 1993; Sainburg et al., 1993; Miall et al., 2018). There was no significant difference in age between each deafferented patient and the control participants (z-score IW = −0.50; z-score GL = 0.28). None of the control participants reported having neurological or sensorimotor disorder. This study was specifically reviewed and approved by the institutional review board of the ISIR, and it was conducted in accordance with its recommendations. All the participants gave their written informed consent in accordance with the Declaration of Helsinki. They were all naive to the purpose of the experiment.

### Stimuli

The four ambiguous lowercase letters b, d, p, and q were manually drawn by the experimenter on the participants’ head surfaces with a rubber tipped stylus pen. The letters were drawn in one continuous stroke, beginning from the stem and ending with the loop. The letters were as close as possible to 5 × 5 cm in size. The experimenter was trained to draw the letters with a constant speed and pressure. The duration for tracing each letter was approximately 2 s. The letters were drawn on the center of the forehead and on the left and right temples. The tactile perception of the two deafferented participants was tested for these head surfaces before the experiment, and they both confirmed perceiving correctly the letters.

### Procedure

Each participant was comfortably seated on a chair during the experiment. On each trial, one of the four letters was drawn on the participants’ surface of the head. The participants were instructed to verbally report the letter they perceived as spontaneously as possible. They were informed that each letter could be recognized in different ways, depending on how they assign the left-right and top-bottom axes of the letter, and that there were consequently no correct or incorrect responses. The reported response was registered by the experimenter before drawing the next letter.

The participants’ head orientation in yaw relative to the trunk varied according to three different conditions: forward (i.e., aligned with the trunk), leftward (i.e., turned toward the left shoulder), and rightward (i.e., turned toward the right shoulder). For the leftward and rightward orientations, the participants were instructed to turn the head as close as possible to a 90° rotation in yaw, without feeling any discomfort. The participant’s head was rotated around 60–70° in yaw relative to the trunk. The degree of head rotation was similar for the two patients and the controls and for the two directions of rotation (i.e., leftward and rightward). All along the experiment, the experimenter corrected the participants’ head position if the rotation they performed did not match the one they achieved in the first set of four trials or if they performed head rotation in roll or in pitch. For each condition, the participants held their head rotated for four consecutive trials (i.e., approximately 20 s, corresponding to the tracing of the four letters plus the participants’ answers). After this delay, the participants were asked to move their head to the next position. The experimenter frequently asked the participants about their fatigue or discomfort and encouraged them to take a break between two conditions whenever they feel tired. Note, however, that neither the control participants nor the patients reported neck fatigue due to the different head positions.

During the session with eyes closed, the participants were asked to close their eyes before turning the head and to keep their eyes closed during the four consecutive trials of each condition. However, they could open their eyes between two conditions. For some participants, the eyes-closed head turning varied relative to eyes open, not only in yaw but also in roll or pitch. In these cases, the experimenter corrected the head position. The degree of head rotation was thus similar in the sessions with eyes closed and open.

### Design

The experiment was divided into two sessions, one with eyes open and the other with eyes closed. The two deafferented patients performed the graphesthesia task with eyes open first and then eyes closed. For the control participants, in order to control for any order effect, half of them began with eyes open, whereas the other half began with eyes closed. Each of the two sessions was divided into two blocks of 36 trials, with a short break in between, resulting in a total of 144 trials for the entire experiment. Note that, due to fatigue, IW has not completed the last session of the experiment (i.e., the second block of trials of the session with eyes closed; he thus completed a total of 108 trials out of 144). In each block of trials, there were nine conditions resulting from the combination of the three head surfaces and the three head orientations. Thus, in each of the two sessions, there were eight trials for each condition (two presentations of each of the four letters). In each block of trials, the four letters were drawn consecutively with the same head surface and head orientation. The order of the nine conditions (3 head surface × 3 head orientation) in one block and the order of the four letters for each of the nine conditions were randomized for each participant.

### Data Analysis

Each of the participants’ responses was categorized as resulting from the adoption of a self-centered perspective (e.g., response d for the letter b from the experimenter’s point of view) or a decentered one (e.g., response b for the letter b). The responses corresponding to vertical inversions (e.g., response p or q for the letter b) represented only 2.7% of trials overall. They were considered as errors and they were excluded from subsequent analyses. After excluding the errors, the proportion of self-centered responses was computed for each participant and each condition. To compare the results of GL and IW with those of control participants, t-test comparisons of a single value to a population sample was used (Nougier et al., 1996; Sarlegna et al., 2010). 95% confidence intervals were also provided.

## Results

### Global Preferences for Self-Centered Versus Decentered Perspectives

**Figure 3:**
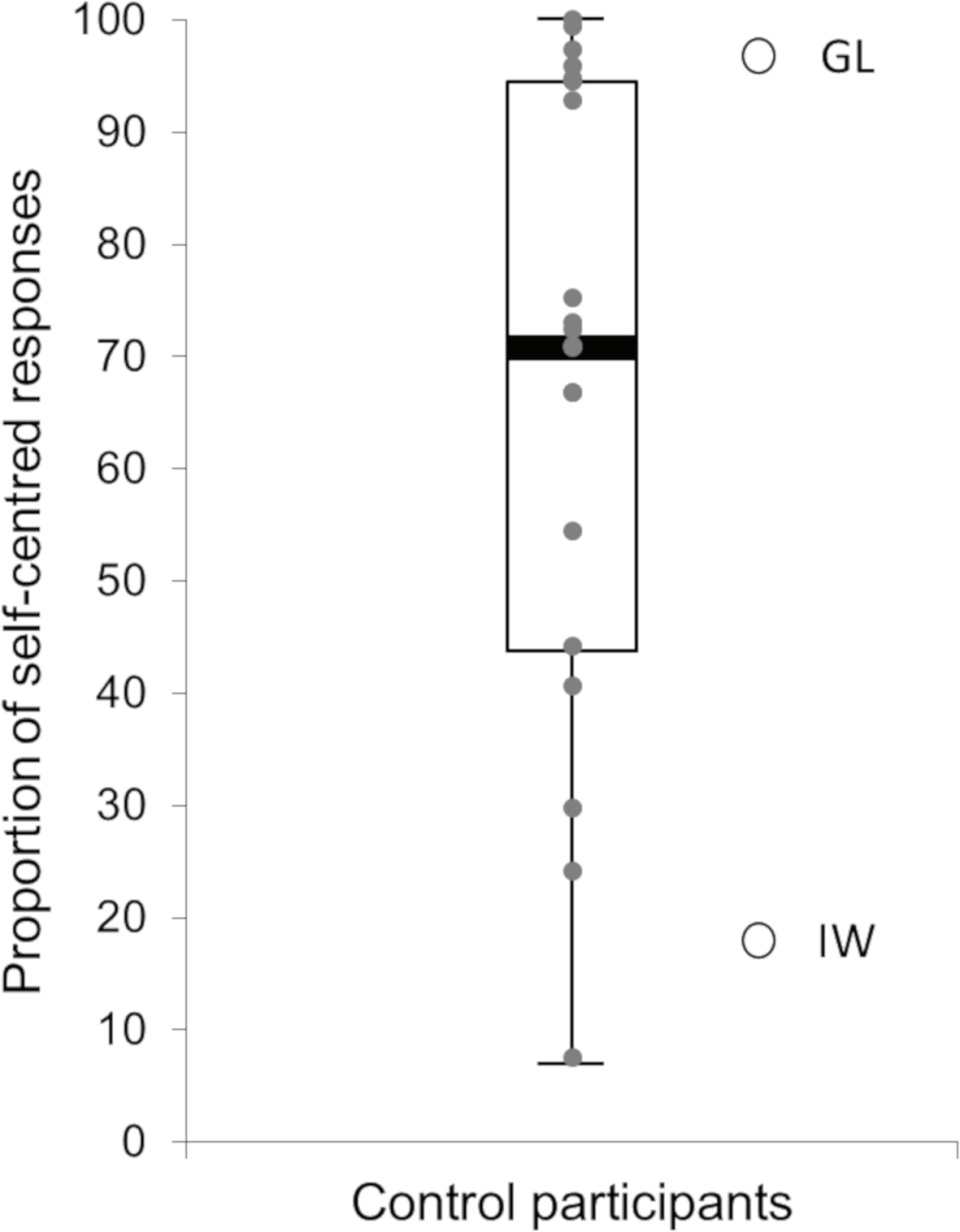
Participants’ global proportion of self-centered responses. The box-and-whisker plot represents control participants’ data. The gray circles represent controls’ individual data. The two white circles represent the two deafferented patients’ data.

All the participants, including the two deafferented patients, felt very well the stimulation (b, d, p, or q) on their forehead and sides of their head. Most of the control participants’ responses corresponded to the adoption of a self-centered perspective (68.3%, *SD* = 39.9). Figure 3 represents the participants’ global proportion of self-centered responses (median = 71.5%, *Q1* = 51.6%, *Q3* = 94.5%, min = 6.9%, max = 100.0%). It shows a clear bias toward the adoption of self-centered perspectives for control participants with, however, an important interindividual variability. Moreover, only five control participants reported decentered responses most of the time (i.e., superior to 50%). Among them, only two participants reported more than 75% of decentered responses. Regarding the two deafferented patients’ responses, GL reported a strong majority of self-centered responses (96.5%, *SD* = 7.2), whereas IW reported most of the time decentered responses (83.3%, *SD* = 15.3). Their proportion of self-centered responses were both significantly different from the control participants’ proportion (*t*(19) = 4.56, *p* < 0.001, *η* ^2^ = 0.523, for GL; *t*(19) = 8.01, *p* < 0.001, *η* ^2^ = 0.772, for IW) and beyond the 95% confidence interval of the control participants’ proportion [95% *CI* = (55.4, 81.3)]. For control participants, the slightly greater proportion of self-centered responses in female (75.3%, *SD* = 25.6) than male (61.3%, *SD* = 29.2) was not significant (*t*(18) = 1.14, *p* = 0.269, *η* ^2^ = 0.067).

### Effects of Body Posture and Vision

To evaluate the effect of body posture on the adoption of self-centered versus decentered perspectives in control participants, an ANOVA was conducted on the proportion of self-centered responses with orientation of the head (forward, leftward, rightward), stimulated surface (forehead, left side, right side), and vision (eyes open, eyes closed) as within-participant factors and order between eyes open and eyes closed as a between-participant factor. There was a significant effect of the stimulated surface [*F*(2,38) = 14.28, *p* < 0.001, *η* ^2^ = 0.429]. The proportion of self-centered responses was significantly greater for the forehead (mean = 84.1%, *SD* = 26.7) than for the two sides of the head [*F*(1,19) = 19.78, *p* < 0.001, *η* ^2^ = 0.510], with no significant differences [*F*(1,19) = 1.34, *p* = 0.262, *η* ^2^ = 0.066] between the left (mean = 62.8%, *SD* = 31.1) and right (mean = 58.1%, *SD* = 33.8) sides of the head.

**Figure 4:**
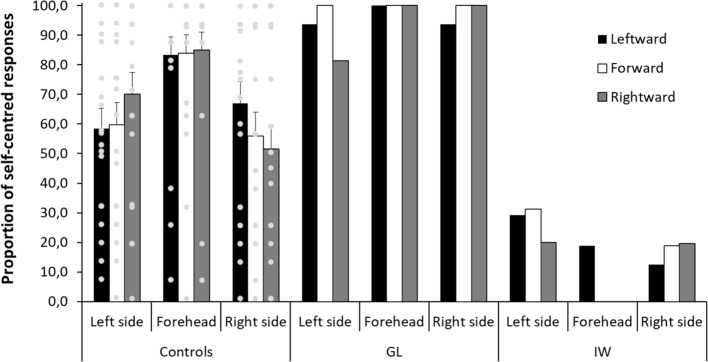
Control participants and patients’ proportion of self-centered responses as a function of the stimulated surface (left side, forehead, right side) and the orientation of the head (leftward, forward, rightward). The gray circles represent the controls’ individual data. Error bars represent the standard errors of the mean for control participants.

Importantly, there was a significant interaction between the orientation of the head and the stimulated surface [*F*(4,76) = 8.87, *p* < 0.001, *η* ^2^ = 0.318]. Figure 4 shows that the proportion of self-centered responses on the forehead was not influenced by the orientation of the head in yaw relative to the trunk (83.9%, *SD* = 27.4, for the head oriented forward; 83.3%, *SD* = 27.5, for the head oriented leftward; 85.0%, *SD* = 27.2, for the head oriented rightward). On the contrary, the proportion of self-centered responses on the sides of the head was influenced by the orientation of the head in yaw relative to the trunk. When the head was oriented forward, the proportion of self-centered responses did not significantly differ from chance level (59.7%, *SD* = 33.8, *t*(19) = 1.28, *p* = 0.216, *η* ^2^ = 0.079, for the left side; 55.9%, *SD* = 36.6, *t*(19) < 1, *ns*, for the right side). When the head was oriented leftward, the proportion of self-centered responses on the right side (67.1%, *SD* = 32.5) was significantly greater than when the head was oriented forward [*F*(1,19) = 7.56, *p* < 0.05, *η* ^2^ = 0.285] and it became significantly superior to chance level [*t*(19) = 2.35, *p* < 0.05, *η* ^2^ = 0.225]. Finally, when the head was oriented rightward, the proportion of self-centered responses on the left side (70.1%, *SD* = 33.1) was significantly greater than when the head was oriented forward [*F*(1,19) = 5.98, *p* < 0.05, *η* ^2^ = 0.240] and it became significantly superior to 50% [*t*(19) = 2.72, *p* < 0.05, *η* ^2^ = 0.280]. The adoption of self-centered versus decentered perspectives in control participants was therefore influenced by the stimulated surface and the body posture. However, Figure 4 also shows that there was an important interindividual variability in the adopted perspective in every condition.

Regarding the deafferented patients, the adoption of self-centered versus decentered perspectives does not appear to be influenced by the orientation of the head relative to the trunk. GL was influenced neither by the orientation of the head nor by the stimulated surface as she almost systematically adopted a self-centered perspective (see Figure 4). More specifically, when the sides of her head were stimulated, she adopted a self-centered perspective, whatever the orientation of her head. Contrary to control participants, for the forward orientation, her proportion of self-centered responses (100%) was well above chance level. It was significantly different from the control participants’ proportion [*t*(19) = 5.67, *p* < 0.001, *η* ^2^ = 0.629] and beyond the 95% confidence interval of the control participants’ proportion [95% *CI* = (42.2, 73.4)]. Thus, contrary to control participants, she adopted a self-centered perspective even when the tactile letters were drawn on a side surface of the head, which was not aligned with the front surface of the trunk.

IW’s results reflect a preference for a decentered perspective. Consequently, Figure 4 shows fewer self-centered responses for him than for GL and control participants. Even though his preference was not as strong and systematic as that of GL, the preference for a decentered perspective was clear for the forehead (93.8%), left (73.2%), and right (83.0%) sides of the head. Importantly, when the head was oriented forward, the proportion of self-centered responses (25.0%) on the sides of the head was significantly different from the control participants’ proportion [*t*(19) = 4.41, *p* < 0.001, *η* ^2^ = 0.506] and was beyond the 95% confidence interval of the control participants’ proportion [95% *CI* = (42.2, 73.4)]. Thus, contrary to control participants and similarly to GL, IW adopted a constant perspective across conditions even when the tactile letters were drawn on a side surface of the head, which was not aligned with the front of the trunk. Taken together, these results show that GL was clearly not influenced by the orientation of her head in yaw relative to her trunk when adopting a self-centered perspective, whereas IW adopted mostly a decentered perspective, with more variability than GL, but without showing the same pattern of responses than control participants.

Finally, there was no significant main effect of vision in control participants (68.9%, *SD* = 26.2, for eyes open; 67.8%, *SD* = 30.1, for eyes closed; *F*(1,19) <1, *ns*), but there was a significant interaction between vision and the orientation of the head [*F*(1,19) = 3.76, *p* < 0.05, *η* ^2^ = 0.165]. This interaction showed a significant effect of vision only when the head was oriented leftward [*F*(1,19) = 5.66, *p* < 0.05, *η* ^2^ = 0.230], with a greater proportion of self-centered responses with eyes open (72.5%, *SD* = 26.2) than with eyes closed (66.7%, *SD* = 29.8), but not when the head was oriented forward [*F*(1,19) = 1.87, *p* = 0.188, *η* ^2^ = 0.089] and rightward [*F*(1,19) <1; *ns*]. Regarding the deafferented patients, GL showed no effect of vision (95%, for eyes open; 97.2%, for eyes closed), whereas IW reported a slightly greater proportion of decentered responses when his eyes were open (86.1%) than when they were closed (80.6%). This difference of 5.5 points of percentage is significantly different from the control participants’ difference [*t*(19) = 2.63, *p* < 0.05, *η* ^2^ = 0.267] and beyond the 95% confidence interval of the control participants’ vision effect [95% *CI* = (−6.4, 4.2)]. This result suggests a greater bias toward adopting the experimenter’s perspective when the experimenter is visible than when he is not.

## Discussion

The present study investigated the role of somatosensory and visual information in the adoption of self-centered versus decentered perspectives. Two deafferented patients (GL and IW) and 20 age-matched control participants performed the graphesthesia task with ambiguous symbols drawn on the forehead, left side, and right side of their head. The orientation of the head in yaw relative to the trunk and the possibility to open or not the eyes were also manipulated to assess the influence of body posture and vision. Regarding control participants, the adoption of a self-centered versus decentered perspective depended on head orientation relative to the trunk. Regarding the deafferented patients, the orientation of the head in yaw relative to the trunk did not influence the adopted perspective, suggesting that somatosensory loss impacts self orientation. Contrary to controls, deafferented patients adopted a self-centered or a decentered perspective even for side surfaces of the head which were not aligned with the front surface of the trunk. Finally, only IW showed a slight effect of vision, with a greater preference for a decentered perspective when the eyes were open than when they were closed, that is, when the experimenter was visible than when he was not. Neither the control participants, nor GL, showed a significant effect of vision.

Self-centered perspectives were adopted in controls for tactile letters drawn on the forehead or on side surfaces of the head which were aligned with the front surface of the trunk. In these conditions, the left-right axis of the tactile letter is aligned with the left-right axis of the head or the trunk. This result confirms the previously reported role of both head and trunk orientations in making spatial judgments relative to the body and the self (Natsoulas and Dubanovski, 1964; Alsmith et al., 2017). The head likely plays a specific role due to the presence of several sensory systems in this body part. The trunk may also be important due to its central place in the body. Head and limb orientation can thus be easily defined relative to the trunk. The transformation of multisensory reference frames into a unified body-centered reference frame, which allows the observer to adopt a unique self-centered perspective on the external world, perceived as being distinct from the self, strongly relies on somatosensory information (see Arnold et al., 2017). For instance, neck proprioception is important to consider the orientation of the head relative to the trunk.

The results obtained with the two deafferented patients clearly show that somatosensory loss impacts the spatial perspectives that are adopted to interpret ambiguous tactile stimulations. Contrary to control participants, the perspective adopted by the patients did not depend on the orientation of their head in yaw relative to their trunk. Although IW has access to proprioceptive information about his neck, which GL has not (Cole and Paillard, 1995), he was not strongly influenced by his head orientation in yaw. However, IW’s access to neck proprioceptive information may explain why his results are more variable than GL’s ones in the graphesthesia task. A possible explanation to this variability is that his global somatosensory loss does not encourage him to use efficiently his preserved neck proprioceptive information. It would be interesting to evaluate further the role of neck proprioception, for instance, with head rotation in roll or in pitch relative to the trunk. As the letters b, d, p, and q are ambiguous not only along the horizontal axis but also along the vertical axis, the graphesthesia task with head rotation in roll relative to the trunk would be particularly interesting as it allows manipulating the vertical head axis relative to both the vertical trunk axis and gravity. With this manipulation, patients might be influenced more by gravity than by body posture, compared to controls.

Our results do not support the hypothesis that deafferented patients rely mostly on a decentered perspective due to a deficit in adopting an egocentric reference frame. While IW mainly adopted a decentered perspective, GL clearly preferred a self-centered one. IW seemed to adopt a strategy based on external information and consisting in taking the experimenter’s perspective, and he confirmed such strategy during a debriefing following the experimental session. IW’s strategy, which relies on imagining how the letter could be seen by the experimenter, is compatible with his great reliance on visual imagery (Ter Horst et al., 2012). On the contrary, GL adopted more an internal strategy, with a systematic choice for a head-centered perspective. Note, however, that GL’s internal strategy may also be visual, as she indicated having mentally projected the letters outside her body, in front of her eyes, during the post-experiment debriefing. Thus, GL’s systematic adoption of a self-centered perspective is compatible with her previously reported dependence for visual information (Bringoux et al., 2016).

Using the graphesthesia task, Ferrè et al. (2014) have shown that self-centered perspectives are mostly adopted when the processes anchoring the self to the body are reinforced, highlighting the important role the body plays in the sense of self. The massive somatosensory loss in deafferented patients has the consequence that the self is less anchored to the body. Thus, self-orientation may rely more on external information, with an important visual dominance, and it may involve more cognitive strategies. During the debriefing, the two patients have indicated having chosen a given perspective that they kept during the entire experiment. Such use of a cognitive strategy may explain the lower variability in their responses than that of control participants. It might be the case that when the information coming from the body is no longer accessible, the sense of bodily self is more thought than felt. This view is consistent with the hypothesis that the body schema, involving a set of motor abilities and habits that enable movements and the maintenance of body posture, is deficient in deafferented patients, whereas the body image, which consists of a set of intentional states and mental representations of one’s own body, is preserved (Gallagher and Cole, 1995).

It remains to understand why the two patients have adopted so different individual strategies. A recent study with these two deafferented patients, investigating their ability to develop and use spatial maps, suggests that individual differences, and thus strategies, may influence their spatial cognition even more than visual or somatosensory signals (Renault et al., 2018). Studies using the graphesthesia task have indicated several perceptual, cognitive, personal, and interpersonal factors that induce individual differences in the adoption of self-centered versus decentered perspectives (see Arnold et al., 2017, for a review). Differences between the two patients may be explained by gender. Males have been reported to adopt more often decentered perspectives than females (Krech and Crutchfield, 1958; Duke, 1966; Deroualle et al., 2017; but see Allen and Rudy, 1970). However, the control participants’ results, similar for the two genders in the present study, do not confirm this gender effect. Some of the two patients’ personality traits may have induced differences in their choice to adopt a self-centered versus a decentered perspective. More generally, the results of both controls and patients in the graphesthesia task show important interindividual variability in the perspective that was overall adopted as well as in the influence of body posture. These results highlight the existence of high-level cognitive processes such as decision criteria or consistency bias, in addition to the lower level perceptual and spatial processes underlying the task. Whereas the latter are influenced by somatosensory information, the former might be similar in deafferented patients and in controls.

To conclude, the present study confirms and extends the previously reported influence of head and trunk orientations in making spatial judgments relative to the body and the self (Natsoulas and Dubanovski, 1964; Alsmith et al., 2017). This result highlights the important role the body plays in perception and self-consciousness. Adopting a self-centered perspective, which is crucial for the multisensory integration underlying self-consciousness, or a decentered one, which is crucial to understand how the world is perceived by other persons, both involve processes that are anchored to the body. When internal information coming from the body is lacking, more cognitive strategies are adopted, based on thinking about the body rather than on feeling it.

## Author Contributions

GA, FS, LF, and MA designed the experiment, performed the statistical analyses, and wrote the paper. GA and LF conducted the experiments.

### Conflict of Interest Statement

The authors declare that the research was conducted in the absence of any commercial or financial relationships that could be construed as a potential conflict of interest.
